# Influence of Human Bone Marrow Mesenchymal Stem Cells Secretome from Acute Myeloid Leukemia Patients on the Proliferation and Death of K562 and K562-Lucena Leukemia Cell Lineages

**DOI:** 10.3390/ijms25094748

**Published:** 2024-04-26

**Authors:** Fábio Alessandro de Freitas, Débora Levy, Cadiele Oliana Reichert, Juliana Sampaio-Silva, Pedro Nogueira Giglio, Luís Alberto de Pádua Covas Lage, Marco Kawamura Demange, Juliana Pereira, Sérgio Paulo Bydlowski

**Affiliations:** 1 Lipids, Oxidation and Cell Biology Team, Laboratory of Immunology (LIM19), Heart Institute (InCor), Medical School of Sao Paulo University (FMUSP), Sao Paulo 05403-900, SP, Brazil; fabio.alessandro@alumni.usp.br (F.A.d.F.); d.levy@hc.fm.usp.br (D.L.); kadielli@hotmail.com (C.O.R.); jukisbio@gmail.com (J.S.-S.); 2 Institute of Orthopedics and Traumatology, Clinic Hospital of Medical School, Sao Paulo University (HCFMUSP), Sao Paulo 05403-010, SP, Brazil; pedrongiglio@gmail.com (P.N.G.); demange@usp.br (M.K.D.); 3 Laboratory of Pathogenesis and Therapy in Onco-Immuno-Hematology (LIM-31), Department of Hematology, Hemotherapy and Cell Therapy, Clinic Hospital of Medical School, Sao Paulo University (HCFMUSP), Sao Paulo 05403-900, SP, Brazil; luis.lage@hc.fm.usp.br (L.A.d.P.C.L.); julianapereira29@hotmail.com (J.P.); 4 National Institute of Science and Technology in Regenerative Medicine (INCT-Regenera), National Council for Scientific and Technological Development (CNPq), Rio de Janeiro 21941-902, RJ, Brazil; 5 Department of General Physics, Physics Institute, Sao Paulo University, Sao Paulo 05508-090, SP, Brazil

**Keywords:** leukemia, bone marrow, mesenchymal stem cells, secretome, cell death, cytokines, ABC transporters

## Abstract

Leukemias are among the most prevalent types of cancer worldwide. Bone marrow mesenchymal stem cells (MSCs) participate in the development of a suitable niche for hematopoietic stem cells, and are involved in the development of diseases such as leukemias, to a yet unknown extent. Here we described the effect of secretome of bone marrow MSCs obtained from healthy donors and from patients with acute myeloid leukemia (AML) on leukemic cell lineages, sensitive (K562) or resistant (K562-Lucena) to chemotherapy drugs. Cell proliferation, viability and death were evaluated, together with cell cycle, cytokine production and gene expression of ABC transporters and cyclins. The secretome of healthy MSCs decreased proliferation and viability of both K562 and K562-Lucena cells; moreover, an increase in apoptosis and necrosis rates was observed, together with the activation of caspase 3/7, cell cycle arrest in G0/G1 phase and changes in expression of several ABC proteins and cyclins D1 and D2. These effects were not observed using the secretome of MSCs derived from AML patients. In conclusion, the secretome of healthy MSCs have the capacity to inhibit the development of leukemia cells, at least in the studied conditions. However, MSCs from AML patients seem to have lost this capacity, and could therefore contribute to the development of leukemia.

## 1. Introduction

Leukemias are one of the most prevalent types of malignant disorders of the hematopoietic cells, characterized by abnormal proliferation and increased lifespan of myeloid or lymphoid cells [1]. Acute myeloid leukemia (AML) is characterized by impaired differentiation and uncontrolled clonal expansion of myeloid progenitors/precursors, resulting in bone marrow (BM) failure and impaired normal hematopoiesis [2]. Moreover, only 40–50% of AML patients can be cured using conventional chemotherapy [3,4].

Hematopoietic stem cells (HSCs) reside in the bone marrow niche, a specialized microenvironment that contains complex and diverse cell populations [5], including the mesenchymal stem cells (MSCs) [6]. These are multipotent cells that contribute substantially to the BM niche [7,8], supporting hematopoiesis as well [9], playing an essential role by regulating HSC proliferation and differentiation. A defect in AML MSCs was shown to lead to dysfunctional crosstalk between the BM niche and HSCs, potentially resulting in AML initiation or propagation [10]. Specific changes in MSCs have also been demonstrated to initiate leukemia in mice [11,12,13]. In addition, it is well accepted that bone marrow MSCs promote leukemia cell resistance to chemotherapy [14].

MSCs may exert their effects on bone marrow cells and surrounding niche via release of extracellular vesicles (EV) such as exosomes or microvesicles or by soluble factors that are responsible for paracrine signaling [15]. MSCs secretome plays crucial roles in maintaining BM homeostasis, such as promoting endogenous repair and tissue regeneration, and contributes to the pathogenesis of diseases as well. It has the potential to alter several aspects of tumor biology, including the proliferative capacity [16,17]. In fact, MSC secretome was shown to inhibit the proliferation of leukemic cells and arrest the cycle of these cells in G0/G1 phase [18]. Exosomes have also been described as modulating ABC transporters [19]. In cancer, ABC transporters are deeply involved in tumor cell resistance to chemotherapy by regulating the flow of anticancer agents into the cancer cells [20].

Here we evaluated the effects of the secretome of BM MSCs derived from acute myeloid leukemia patients and from healthy donors on two leukemia cell lines: (1) K562, a cell lineage established from the pleural effusion of a patient in blast crisis of chronic myeloid leukemia [21]; (2) K562-Lucena, a multidrug resistance (MDR) cell selected by the treatment of K562 cells with increasing concentration of vincristine [22]. Cell proliferation, viability and death were evaluated, together with cell cycle and modulation of cytokine and ABC transporter profile.

## 2. Results

### 2.1. Cell Death Assays

**Figure 1:**
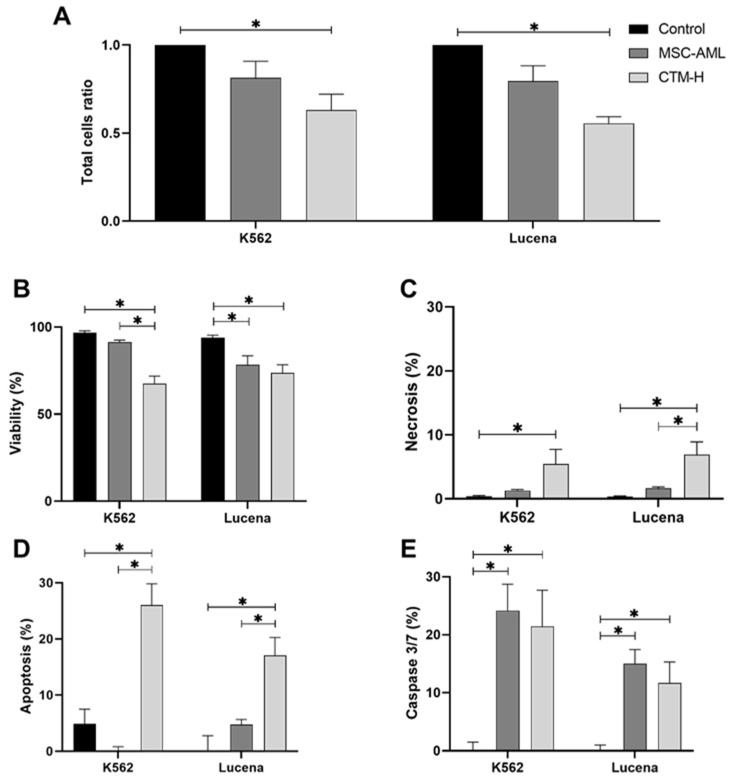
Total cell ratio (**A**), viability (**B**), necrosis (**C**), apoptosis (**D**) and caspase 3/7 activity (**E**) in K562 and Lucena cells after transwell culture by 48 h with bone marrow-derived mesenchymal stem cells from patients with acute myeloid leukemia (MSC-AML) or from healthy donors (MSC-H). Data are expressed as mean ± SEM from at least three independent experiments in duplicate. (*) *p* values ≤ 0.05.

Total cell count ratio decreased in K562 transwell cultured with MSC-H when compared with the control (*p* = 0.015), but not when compared with transwell culture with MSC-AML (Figure 1A). Lucena cells exhibited the same behavior (MSC-AML vs. control, *p* < 0.001) (Figure 1A).

Cell viability decreased in both K562 and Lucena cells transwell cultured with MSC-AML or MSC-H (Figure 1B).

Cell death was also affected by coculturing K562 or Lucena cells with MSC-H. No necrosis was observed in control cells. However, the level of necrosis in K562 and Lucena cells was increased by coculturing with MSC-H (*p* = 0.045 and 0.003, respectively), but not with MSC-AML (Figure 1C).

K562 and Lucena cells showed low apoptosis rates. Again, apoptosis levels increased when these cells were transwell cultured with MSC-H (*p* < 0.001), but not with MSC-AML (Figure 1D).

Caspase 3/7 activation rates were very low in both K562 and Lucena cells. Interestingly, caspase 3/7 activation rates increased in K562 cells transwell cultured with MSC-AML (*p* = 0.002) and MSC-H (*p* = 0.005). The same was observed in Lucena cells transwell cultured with MSC-AML (*p* = 0.003) and MSC-H (*p* = 0.017) (Figure 1E).

**Figure 2:**
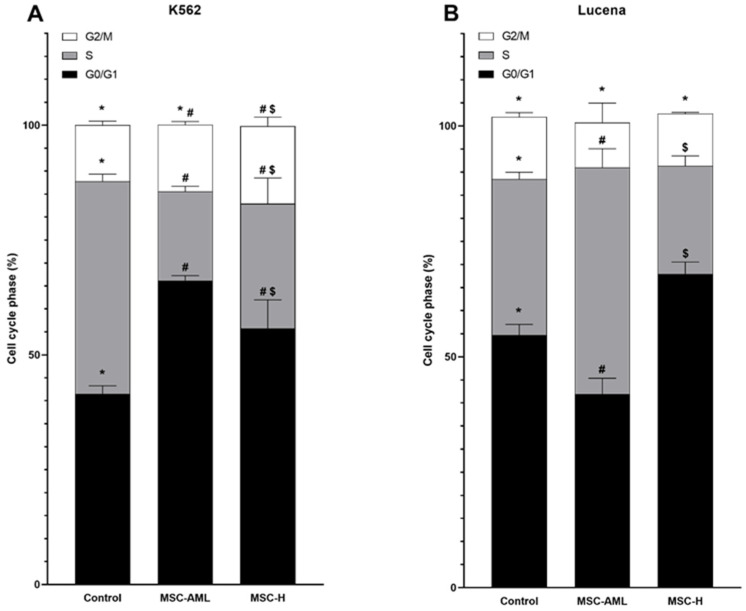
Cell cycle phases of K562 (**A**) and Lucena (**B**) cells after coculturing with mesenchymal stem cells from acute myeloid leukemia patients (MSC-AML) or mesenchymal stem cells from healthy donors (MSC-H) for 48 h. Data are expressed as mean ± SEM from 3 independent experiments in duplicate. Significant *p* values were considered as ≤0.050. Different symbols (*, # or $) in same cell cycle phases indicate statistical significancy.

Figure 2 shows the cell cycle pattern of K562 and Lucena cells transwell cultured with MSC-AML or MSC-H for 48 h. In K562 cells, when compared with control cells, G0/G1 phase increased in both MSC-AML (*p* < 0.001) and MSC-H (*p* = 0.023) transwell cultures. The S phase decreases in both MSC-AML (*p* < 0.001) and MSC-H (*p* = 0.001) transwell cultures. G2/M phase showed alterations in MSC-H (*p* = 0.036) transwell culture, but not in MSC-AML. In Lucena cells, we observed a different behavior. G0/G1 phase decreased in MSC-AML transwell culture (*p* = 0.001) and increased in MSC-H transwell culture (*p* = 0.008). The S phase increased in MSC-AML transwell culture (*p* = 0.002) and decreased in MSC-H transwell culture (*p* = 0.028). No changes were observed in the G2/M phase.

### 2.2. Molecular Biology Assays

**Figure 3:**
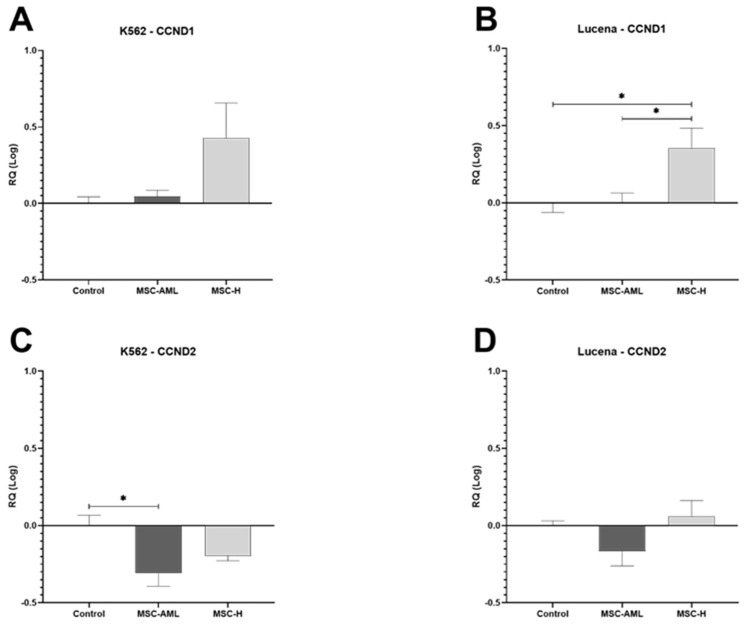
Gene expression of cyclin D1 (*CCND1*) and cyclin D2 (*CCND2*) in K562 and Lucena cells after transwell culture by 48 h with bone marrow-derived mesenchymal stem cells from patients with acute myeloid leukemia (MSC-AML) or from healthy donors (MSC-H). (**A**) K562 gene expression of *CCND1*; (**B**) Lucena gene expression of *CCND1*; (**C**) K562 gene expression of *CCND2*; (**D**) Lucena gene expression of *CCND2*. Data are expressed as mean ± SEM from 3 independent experiments in duplicate. (*) *p* ≤ 0.05.

*CCND1* gene expression in K562 control cells did not change by coculturing with MSC-AML or MSC-H (Figure 3A). *CCND1* expression in Lucena cells, however, increased only after coculturing with MSC-H (*p* = 0.017) (Figure 3B).

*CCND2* gene expression in K562 cells transwell cultured with MSC-AML decreased (*p* = 0.011) but not after MSC-H transwell culture (Figure 3C). *CCND2* gene expression in Lucena cells was not affected by coculturing with MSC-AML or MSC-H (Figure 3D).

**Figure 4:**
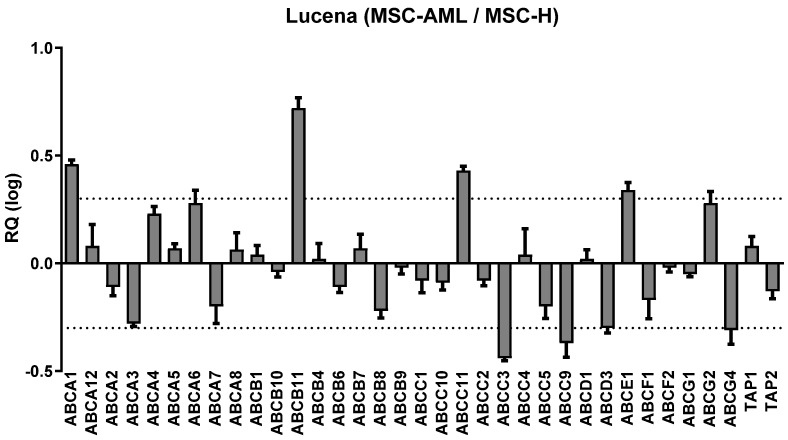
ABC transporters gene expression of Lucena cells transwell cultured with mesenchymal stem cells from healthy donors (MSC-H) compared with Lucena cells transwell cultured with mesenchymal stem cells from acute myeloid leukemia patients (MSC-AML). Results were obtained from a pool of cDNA samples from 3 independent experiments. Data are expressed as mean ± SEM. Dotted lines are the cutline to overexpression (RQ Log = 0.30) or inhibition (RQ Log = −0.30) of ABC transporter gene expressions. RQ: relative quantification. Dotted lines represent the significance limits of RQ (relative quantification) changed by at least two-fold: more than 2 (RQ log = 0.30) or less then 0,5 (RQ log = −0.30).

Figure 4 shows the gene expression of ABC transporters in Lucena cells transwell cultured with MSC-H, compared to coculturing with MSC-AML. *ABCA1*, *ABCB11*, *ABCC11* and *ABCE1* genes which were overexpressed, while *ABCC3*, *ABCC9*, *ABCD3* and *ABCG4* gene expression was downregulated.

### 2.3. Cytokine Array Assay

Table 1 shows that, from the 39 cytokines tested, only IL-8 was detected in K562 and Lucena cells. In MSC-AML and MSC-H, 6 were observed: CCL2/MCP-1, CXCL12/SDF-1, IL-6, IL-8, MIF and Serpin E1/PAI-1.

Table 2 shows the cytokines detected in K562 and Lucena cells transwell cultured with MSC-AML or MSC-H. In K562 cells, there was an increase in CCL2/MCP-1, and a decrease in IL-6 and IL-8, in cells transwell cultured with MSC-H compared with those transwell cultured with MSC-AML. In Lucena cells, there was an increase in CCL2/MCP-1, IL-6, IL-8 and Serpin E1/PAI-1, and a decrease in CXCL12/SDF-1 in cells transwell cultured with MSC-H compared with those transwell cultured with MSC-AML.

## 3. Discussion

The secretome of MSCs have a paracrine action promoted by structures, such as extracellular vesicles, and soluble factors, such as cytokines [23]. Relatively very few studies have been done on the effects of MSCs and their secretome on leukemia cell proliferation and death. Human umbilical cord blood-derived MSCs were described to inhibit the K562 cell proliferation, together with alterations in cell cycle [24]. Human Wharton’s jelly stem cell secretions were shown to be capable of decreasing the number of K562 cells in vitro by inducing cell cycle arrest and increasing apoptosis [25]. On the other hand, exosomes from human umbilical cord MSCs were described to have no effect on K562 cell viability and apoptosis [26].

Here we have shown that MSC-H secretome acts on leukemic cell lineage K562 by reducing the cell number and viability, while increasing cell death by necrosis and apoptosis. Moreover, the same was observed with the MDR cell lineage Lucena, showing that MSC-H could act on both sensible and resistant leukemic cells. Maybe more importantly, MCS-AML was not able to promote apoptosis or necrosis in both leukemic K562 and Lucena cells.

An increase in caspase 3/7 activation in K562 and Lucena cells transwell cultured with MSC-AML or MSC-H was observed. Activation of caspase 3/7, as expected, was related with increased apoptosis levels in K562 and Lucena cells transwell cultured with MSC-H. However, K562 and Lucena cells transwell cultured with MSC-AML have also increased caspase 3/7 activation with no changes in apoptosis levels. It is known that some cells can survive to caspase activation when the stimulus is transitory or sublethal [27]. This process is called anastasis and can protect cells from permanent damage after exposure to a damaging agent such as chemotherapy and even radiotherapy [28]. Thus, it is possible that MSC-AML secretome has some factors different to that produced by MSC-H that leads leukemic cells to apoptosis. In this way, MSC-AML would be protecting leukemic cells from death or loss of the propriety to combat these cells.

Transwell culture of K562 or Lucena cells with MSC-H promoted cell cycle arrest in the G0/G1 phase and a decrease in the S phase. These findings could be related to increased levels of apoptosis and a decrease in cell proliferation, as described. Anticancer effects of several drugs have been described to promote G0/G1 cell cycle arrest and apoptosis such as 2,4-dinitrobenzenesulfonamide derivative in acute leukemia cells [29], TH-39 in K562 cells [30], Mere 15 in K562 cells [31] and thio-Cl-IB-MECA in HL-60 cells [32].

D-Cyclins 1 and 2 (*CCND1*, *CCND2*) are key elements in the control of cell cycle progression from G1 to S phases [33,34]. It has been described that *CCND1* overexpression is related with the arresting of cell proliferation [35]. Moreover, overexpression of *CCND2* has been associated with high proliferation of leukemic cells (K562 and Lucena) [36], and downregulation of *CCND2* with the arresting of cell proliferation [37].

We have found overexpression of *CCND1* in Lucena cells transwell cultured with MSC-H. In addition, MSC-AML promotes no changes in *CCND1* expression in K562 and Lucena cells. We found also a significative downregulation of *CCND2* expression in K562 cells transwell cultured with MSC-AML. It is possible that *CCND1* overregulation is the mechanism involved in Lucena proliferation arrest, while *CCND2* downregulation is involved in K562 proliferation arrest.

In humans, ABC transporters are expressed in several tissues. Besides their important physiological roles, ABC transporters participate in the process of tumor cell resistance to chemotherapy by regulating the flow of anticancer agents into the cancer cells [20].

Here we have found that some ABC transporter expressions were changed in Lucena cells after coculturing with MSC-H. Overexpression was observed in four ABC transporters: *ABCA1*, *ABCB11*, *ABCC11* and *ABCE1*. Under expression was observed in four other ABC transporters: *ABCC3*, *ABCC9*, *ABCD3* and *ABCG4*.

*ABCA1* deficiency can accelerate myeloproliferative disorder [38]; therefore, the overexpression of *ABCA1* could be involved in the growth suppression of leukemic cells transwell cultured with MSC-H [39]. *ABCC11* is considered a drug efflux pump for nucleotide analogs [40,41], although, *ABCC11* can be associated with fluoropyrimidine resistance in chronic lymphocytic leukemia [40]. In addition, *ABCE1* may play a role in the biological processes of leukemic cells participating in the MDR phenotype of these cells such as the resistance to adriamycin in K562 cells [42].

An overexpression of *ABCC3* was found in patients in blast crisis of chronic myeloid leukemia with disease recurrence [43], and *ABCC3* can promote imatinib efflux being responsible for the failure in imatinib-target treatments [44]. *ABCC3* is considered a potential therapeutic target in human cancers [45] and its downregulation here described may explain in part our results. Trojani et al. (2019) described that *ABCD3* is significantly under-expressed in the chronic phase of chronic myeloid leukemia in patients after 12 months of treatment with nilotinib [46]. The mechanism of drug resistance by *ABCG4* probably involves alterations in the pH value around cancer cells [47]. The under-expression of *ABCC3*, *ABCC9*, *ABCD3* and *ABCG4* in Lucena cells transwell cultured with MSC-H could inhibit some resistance mechanisms developed by these cells and support the action MSC-H secretome in the attempt to eliminate these leukemic cells.

In both K562 and Lucena cells transwell cultured with MSC-AML and MSC-H, the production of six distinct cytokines was observed. 

CCL2/MCP-1, CXCL12/SDF1, IL-6, IL-8, MIF and PAI-1/serpin E1. We have observed a significant elevation in the levels of CCL2/MCP-1 in both MSC-H transwell cultures (K562 and Lucena) when compared with MSC-AML. Little is known about the role of CCL2 in leukemia biology [48]; however, high levels of CCL2 can be associated with a better prognosis in some tumor types such as melanoma [49,50] and pancreatic cancer [51]. In addition, alterations in CCL2/MCP-1 production in leukemic cells can suppress cell proliferation arresting cells in G1 phase of cell cycle mediated by CCND1 [52].

CXCL12/SDF-1, produced by K562 cells, was not affected by coculturing with MSC-AML and MSC-H. Nonetheless, there was a significant decrease in CXCL12/SDF-1 levels in the transwell culture of MSC-H with Lucena cells compared with MSC-AML transwell culture. CXCL12 produced by bone marrow MSCs is a ligand to CXC chemokine receptor 4 (CXCR4), which is highly present on leukemic cells, and is responsible for growth-promoting and anti-apoptotic signals in these cells [53]. In addition, the CXCL12 decrease can lead leukemic cells to be more sensitive to treatment by getting these cells out of the quiescent state [54]. 

IL-6 also can participate as a cell growth factor, as in malignant plasma cells [55]. Thus, the inhibition of IL-6 can eliminate sensitive IL-6 clones of malignance cells creating free tumor environment niches, although favoring the proliferation of other malignant clone cells whose growth is triggered by other growth factors [55].

IL-8 production by BM-MSC can be stimulated by acute myeloid leukemia cells exosomes, contributing to the drug resistance of these cells [56]. Wu at al. (2021) described that both IL-6 and IL-8 levels are increased in resistant acute myeloid leukemia cells [57]. Thus, IL-8 could promote proliferation and resistance in acute myeloid leukemia cells, while IL-6 predicts survival rate and regulates the drug resistance in these cells [57].

Interestingly, we have a found significant reduction in IL-6 and IL-8 levels in the transwell culture of MSC-H with K562 cells compared with the transwell culture with MSC-AML. Nonetheless, we have observed increased IL-6 and IL-8 levels in Lucena cells transwell cultured with MSC-H in comparison with the transwell culture with MSC-AML.

MIF is secreted by several cells, including MSC [58] and K562 cells [59]. Liu et al. (2020) described that MIF is present in exosomes of bone marrow MSCs, promoting paracrine actions, acting on the maintenance of MSC survival and rejuvenation [60].

Serpin E1/PAI-1 is a negative regulator of MSC survival [61], and curiously is significantly reduced in the Lucena/MSC-AML transwell culture.

Bone marrow MSCs and their secretome are key components in leukemia bone marrow microenvironment, playing critical roles in leukemia progression [62]. In addition, it is known that there is a modulation on cytokine profiles by bone marrow MSCs in leukemia that favors leukemia maintenance and development [63]. Here we have shown that transwell culturing leukemic cells (K562 and Lucena) with MSC-H and MSC-AML promotes alterations on cytokine profiles. How these changes would affect the behavior of leukemia cells remains to be investigated.

## 4. Materials and Methods

This study was conducted in accordance with the Declaration of Helsinki (1975, revised in 2013), and the protocol was approved by the Ethics Committee of the Institution (CAAE: 24060619.0.0000.0068 of 27 November 2019).

### 4.1. Cell Culture and Transwell Culture

#### 4.1.1. Cell Lineages Culture

Two human leukemic cell lineages were used: K562 (ATCC CCL-243), a cell line derived from chronic myeloid leukemia (in blast crisis) and vincristine sensible; and K562-Lucena (Lucena) (BCRJ Code 0127), a MDR cell lineage kindly donated by Dr Vivian Rumjanek. Cells were grown in 182 cm^2^ culture flasks (Santa Cruz Biotechnology, Dallas, TX, USA) with Dulbecco’s Modified Eagle Medium (DMEM) (Sigma-Aldrich, Saint Louis, MO, USA) supplemented with 10% heat-inactivated fetal bovine serum (FBS) (Vitrocell, Waldkirch, Germany) and 1% antibiotics (streptomycin (100 µg/mL) and penicillin (100 UI/mL), Sigma-Aldrich, Germany), incubated at 37 °C in a 5% CO_2_ atmosphere (Thermo Scientific™ Forma™ Series II, Thermo Fisher Scientific, Marietta, OH, USA), until experiments were performed. MDR phenotype of Lucena cells was maintained by the addition of vincristine (60 nM; Sigma-Aldrich, Deisenhofen, Germany). Both leukemic cells were adapted to grow in DMEM medium, rather than in RPMI, by at least 20 consecutive passages with this medium. No changes in proliferation or death rates were observed. This was a necessary step because the culture of MSCs is in DMEM media. Cell lineages were evaluated by STR profile. 

Human bone marrow MSCs were obtained from 3 acute myeloid leukemia patients (MSC-AML) and from 3 healthy donors (MSC-H), after written informed consent. These cells were from the laboratory biorepository and were cultured as described above for K562 cells. These cells were characterized as MSCs as previously described [64].

#### 4.1.2. Transwell Cultures

MSC-AML or MSC-H were seeded (2.0 × 10^5^ cells/well) on 6-well flat bottom polystyrene microplates (Corning, Kennebunk, ME, USA) and incubated at 37 °C in a 5% CO_2_ atmosphere for 24 h. Medium was then removed and 3 mL of DMEM supplemented with 10% FBS, and 1% antibiotics was added in each well. PET membrane with 0.4 µm pores (Corning^®^ Costar^®^ Transwell^®^, Kennebunk, ME, USA) was inserted in each well and 1.0 × 10^6^ cells (K562 or Lucena), suspended in 1.0 mL of the same medium, were added inside each transwell and cultured for 48 h in the same described conditions. Control group refers to K562 or Lucena cells seeded in the same conditions with no addition of MSCs.

### 4.2. Cell Death Assays

#### 4.2.1. Total Cell Count and Cell Viability Assay

K562 or Lucena cells (1.0 × 10^4^) from the above experiments were transferred to 96-well black flat bottom polystyrene microplates. PBS was added to complete 100 µL volume before incubation with 0.1 µg/mL Hoechst 33342 (Molecular Probes, Eugene, OR, USA) and 100.0 µg of propidium iodide (PI) (Molecular Probes, Eugene, OR, USA) for 15 min. The ImageXpress Micro High Content Screening System (Molecular Devices, San José, CA, USA) was used to determine the total cell count and the number of live and dead cells. Five sites per well and 2 wells per condition were acquired. Cell Scoring MetaXpress software (version 5.0, Molecular Devices, San José, CA, USA) was used to analyze the number of cells and the viability. Total cell ratio was obtained by dividing transwell cultured cell count values by their respective cell control count values.

#### 4.2.2. Apoptosis and Necrosis

1.0 × 10^4^ of K562 or Lucena cells (control or transwell cultured with MSC-AML or MSC-H) were transferred to a 96-well plate and the volume was completed to 100 µL with PBS. The Annexin V: FITC Apoptosis Detection Kit I (BD Biosciences, San Jose, CA, USA) was used to determine the percentage of apoptotic and necrotic cells as described by the manufacturer. The nuclei were counterstained with 0.1 µg/mL Hoechst 33342. Apoptosis was determined using an ImageXpress and the MetaXpress software Five sites per well and two wells per condition were acquired. Cells stained with Hoechst 33342 were considered as living cells. An apoptotic process was defined by the presence of Hoechst 33342 and Annexin V or Annexin V/PI. A necrotic process was defined by the presence of Hoechst 33342 and PI.

#### 4.2.3. Detection of Caspase 3/7 Activity

A total of 1.0 × 10^4^ of K562 or Lucena cells (control or transwell cultured with MSC-AML or MSC-H) were transferred to a 96-well plate and the volume was completed to 100 µL with PBS. Caspase 3/7 activity was measured after transferring using CellEvent Caspase 3/7 Green (Invitrogen Life Technologies, Carlsbad, CA, USA) as described by the manufacturer. The nuclei were counterstained with 0.1 µg/mL Hoechst 33342. Fluorogenic substrates were determined using the ImageXpress and the MetaXpress software. Five sites per well and two wells per condition were acquired.

#### 4.2.4. Cell Cycle Analysis

A total of 1.0 × 10^6^ K562 or Lucena, cells (control or transwell cultured with MSC-AML or MSC-H) were collected and fixed with cold 70% ethanol and stored at −20 °C until experiments were performed. Cells were washed, resuspended in PBS and incubated at 37 °C for 45 min with 100 µg/mL RNAse and 20 µg/mL PI. Flow cytometric analysis was performed in FACSCantoTM II flow cytometer (Becton Dickinson, San Jose, CA, USA) and the percentage of DNA content in the different cell cycle phases was determined using FlowJoTM software v10.5.3 (Becton Dickinson, Ashland, OR, USA).

### 4.3. Molecular Biology Assays

A total of 1.0 × 10^6^ of K562 or Lucena cells (control or transwell cultured with MSC-AML or MSC-H) were washed with PBS, 1.0 mL of TRI Reagent^®^ (Sigma-Aldrich, St. Louis, MO, USA) was added and RNA was extracted as described by the manufacturer. RNA was resuspended in 20 µL of DEPC-treated water and spectrophotometrically quantified with NanoDrop 1000 Spectrophotometer (Thermo Fisher Scientific, Wilmington, DE, USA). For cDNA synthesis, 1 µg of RNA was incubated with RQ1 RNase-Free Dnase (Promega, Madison, WI, USA) as described by the manufacturer. This step was followed by cDNA synthesis, using the High-Capacity cDNA Reverse Transcription kit (Applied Biosystem, Waltham, MA, USA) as described by manufacturer.

#### 4.3.1. Cyclin D1 (CCND1) and Cyclin D2 (CCND2) Gene Expression

The 7500 Fast Real-Time PCR System (Applied Biosystems, Waltham, MA, USA) was used to evaluate the expression of genes *CCND1* and *CCND2* from K562 and Lucena cells. TaqMan hydrolysable probes for *CCND1* (Hs00765553_m1) and *CCND2* (Hs00153380_m1) and TaqMan Universal Master Mix were purchased from Applied Biosystems. The expression of mRNA was normalized to glyceraldehyde-3-phosphate dehydrogenase (*GAPDH*, 402869, Thermo Fisher, Waltham, MA, USA), using the comparative cycle threshold (CT); duplicates did not exceed a 0.5 CT value. Tests were performed using the 2^−ΔΔCT^ method [65].

#### 4.3.2. ABC Transporter Gene Expression

The expression of 44 ABC transporters was measured in cDNA samples from Lucena cells transwell cultured with MSC-AML (reference) or MSC-H, using the TaqMan^®^ Array 96 Well FAST Plate Human ABC Transporters (Applied Biosystems, Life Technologies Corporation, Pleasanton, CA, USA) and the 7500 v.2.0.5 software. Significance was achieved when RQ (relative quantification) was changed by at least two-fold: more than 2 (RQ log = 0.30) or less then 0.5 (RQ log = −0.30) [66].

### 4.4. Cytokine Array Assay

The supernatants from isolated cultures of K562, Lucena, MSC-AML and MSC-H or transwell cultures of K562 or Lucena cells with MSC-AML or MSC-H were collected, filtered through 0.22 µm membrane and stored at −80 °C until analysis was performed. The production of 36 cytokines was evaluated in a pool of supernatant of each condition using the semiquantitative kit Proteome Profiler Antibody Arrays Human Cytokine Array (R&D Systems, Minneapolis, NE, USA), according with the manufacturer instructions. The images were obtained with ImageQuant LAS 4000 (GE Healthcare Bio-Sciences AB, Uppsala, Sweden) adjusted to chemiluminescence, after 10 min of exposure, and analyzed in Image Quant TL 8.1 software (GE Healthcare Bio-Science, Piscataway, NJ, USA) in array analysis mode. Data were normalized by dividing values by the mean of positive controls and multiplying by 1000.

### 4.5. Statistical Analysis

Results are expressed as mean ± SEM from at least three independent experiments of each sample. Means were compared using ANOVA followed by the Tukey post-hoc test or by Student’s *t* test when adequate. Analysis was performed in GraphPad Prism version 9.3.0 (GraphPad Software, La Jolla, CA, USA). *p* values ≤ 0.05 were considered significant.

## 5. Conclusions

Leukemic cells are influenced by the secretome profiles of bone marrow mesenchymal stem cells, creating a niche which is somehow favorable for disease progression.

Healthy MSC secretome can inhibit cell growth, reduce cell number and viability, while increasing cell death by necrosis and apoptosis in the leukemic cell lines K562 and Lucena, showing that MSC-H could act on both sensible and resistant leukemic cells. These effects are aligned with alterations in ABC transporters expression, cell cycle arrest in G0/G1 phase and decrease in S phase, and alterations in cytokine profile. Maybe more importantly, the MCS-AML secretome was not able to promote apoptosis or necrosis in both leukemic K562 and Lucena cells, showing that MSC-AML may be permissive to leukemia development. 

## Figures and Tables

**Table 1:** Cytokine detection in K562, Lucena, MSC-AML and MSC-H.

Cytokine	K562	Lucena	MSC-AML	MSC-H
CCL2/MCP-1	-	-	24.03 ± 0.44	4.41 ± 0.16
CXCL12/SDF-1	-	-	4.16 ± 3.09	4.15 ± 0.71
IL-6	-	-	7.87 ± 4.73	20.60 ± 0.54
IL-8	4.45 ± 0.09	3.30 ± 0.50	8.67 ± 0.36	3.23 ± 0.21
MIF	-	-	2.93 ± 1.22	2.72 ± 0.45
Serpin E1/PAI-1	-	-	39.28 ± 5.27	39.11 ± 2.71

MSC-AML: mesenchymal stem cells from acute myeloid leukemia patients; MSC-H: mesenchymal stem cells from healthy donors; CCL2: C-C motif chemokine ligand 2; MCP-1: monocyte chemoattractant protein 1; CXCL12: C-X-C motif chemokine 12; SDF-1: stromal cell-derived factor 1; IL-6: interleukin 6; IL-8: interleukin 8; MIF: macrophage migration inhibitory factor; PAI-1: plasminogen activator inhibitor-1; -: not detected. Data are shown as pixels (mean ± SEM).

**Table 2:** Cytokine production in K562 and Lucena cells transwell cultured with MSC-AML and MSC-H.

Cytokine	K562			Lucena		
	MSC-AML	MSC-H	*p*	MSC-AML	MSC-H	*p*
CCL2/MCP-1	22.8 ± 2.6	113.5 ± 11.4	0.016	17.5 ± 0.7	78.5 ± 5.0	0.007
CXCL12/SDF-1	5.1 ± 1.1	8.5 ± 0.1	0.082	4.4 ± 0.1	3.1 ± 0.1	0.012
IL-6	83.1 ± 2.8	65.4 ± 1.1	0.027	84.5 ± 6.3	128.5 ± 1.5	0.021
IL-8	28.7 ± 2.4	15.8 ± 0.1	0.033	23.9 ± 2.3	43.0 ± 2.2	0.027
MIF	27.5 ± 1.8	32.8 ± 1.8	0.167	22.6 ± 1.1	20.8 ± 1.1	0.369
Serpin E1/PAI-1	172.9 ± 5.1	188.7 ± 20.9	0.540	136.8 ± 2.6	182.1 ± 2.3	0.006

MSC-AML: mesenchymal stem cells from acute myeloid leukemia patients; MSC-H: mesenchymal stem cells from healthy donors; CCL2: C-C motif chemokine ligand 2; MCP-1: monocyte chemoattractant protein 1; CXCL12: C-X-C motif chemokine 12; SDF-1: stromal cell-derived factor 1; IL-6: interleukin 6; IL-8: interleukin 8; MIF: macrophage migration inhibitory factor; PAI-1: plasminogen activator inhibitor-1. Data are shown as pixels (mean ± SEM).

## Data Availability

The datasets generated during the current study are not publicly available but are available from the corresponding author on reasonable request.
